# *In*-*situ* Pb^2+^ remediation using nano iron particles

**DOI:** 10.1186/s40201-015-0157-3

**Published:** 2015-01-21

**Authors:** Mohammad Reza Fadaei Tehrani, Abolfazl Shamsai, Manoochehr Vossughi

**Affiliations:** Department of Civil Engineering, Sharif University of Technology, Tehran, Iran; Institute of Biotechnology and Environment (IBE), Department of Chemical Engineering, Sharif University of Technology, Tehran, Iran

**Keywords:** nZVI, *in*-*situ*, Remediation, Lead, Bench-scale

## Abstract

Originally, application of nano zero valent iron (nZVI) particles for the removal of lead (Pb^2+^) in porous media was studied. At first, stabilized nZVI (S-nZVI) was prepared and characterized, then used in batch and continuous systems. Based on the batch experiments, corresponding reaction kinetics well fitted with the pseudo-first-order adsorption model, and reaction rate ranged from 0.01 to 0.04 g/mg/min depend on solution pH and the molar ratio between Fe and Pb. In batch tests, optimal condition with more than 90% removal efficiency at 60 min was observed at a pH range of 4 to 6 and Fe/Pb ratio more than 2.5. Continuous experiments exposed that Pb^2+^ remediation was as well influenced by seepage velocity, grain size, and type of porous media. The maximum Pb^2+^ removal efficiency in batch and bench-scale systems were 97% and 81%, correspondingly. The results have shown the ability of S-nZVI to use in permeable reactive barriers, as an efficient adsorbent for Pb^2+^, because of its excellent stability, high reducing power, and a large surface area.

**Electronic supplementary material:**

The online version of this article (doi:10.1186/s40201-015-0157-3) contains supplementary material, which is available to authorized users.

## Background

During the last two decades, presence of heavy metal ions in the environment, especially in water sources, was becoming a major concern due to their non-biodegradability, toxicity, wide-spread presence, and tendency to accumulate in living organisms [1]. Lead, a main concern metal pollutant, is widely used in battery manufacturing, electroplating industry, painting and printing processes, plumbing and the combustion of automobile petrol [2]. The U.S. Environmental Protection Agency (U.S.EPA) has set a permissible limit of 0.015 mg/L in drinking water and has placed it on top of the priority list of toxic pollutants [3]. Lead pollution can cause nervous system damage, renal kidney disease, mental retardation, cancer, and anemia in humans [2]. Chemical reduction, ion exchange, chemical precipitation, mineral adsorption, membrane separation, and bio-sorption are the most frequently used treatment technologies for Pb^2+^ removal [4] which are *ex*-*situ* techniques. Most of these methods are only suitable for the removal of Pb^2+^ in low concentrations and often require extensive processing as well as being too expensive.

Recently, *in*-*situ* techniques such as permeable reactive barriers (PRB) have become promising alternatives to *ex*-*situ* methods owing to their lower operating costs [5]. Nano zero valent iron particles could be used as reducing agents in PRBs for removing the wide range of pollutions that promises to be significantly more effective than granular iron, the reaction rates are 25 to 30 times faster, and the sorption capacity is much higher compared with granular iron [2].

The high reactivity of nZVI is the consequence of greater total surface area, higher density of reactive sites on the particle surface, and/or more intrinsic reactivity of the surface sites [6]. Iron nano particles have been extensively studied to remediate pollutants such as chlorinated compounds and metal ions [7], nitrate [8], carbon tetrachloride, benzoquinone [9], metalloids such as arsenic [10], and organic compounds [11]. However, there are many uncertainties regarding to the features of nZVI-based remediation technologies, which have made it difficult to engineer applications for optimal performance or to assess the risk to human or ecological health. In this study, application of surface modified nZVI (S-nZVI) for Pb^2+^ remediation in porous media was experimented that consists of following steps: (1) synthesis, stabilization, and characterization of S-nZVI; (2) determination of kinetics of Pb^2+^ removal by nZVI and the key factors affecting the reaction; (3) investigation of the effects of flow characteristics on the removal rate; (4) bench-scale modeling of lead remediation under natural conditions.

## Methods

### Materials

Lead nitrate (Pb(NO_3_)_2_), used as the source of Pb^2+^ in all experiments, and other chemical reagents, including FeCl_3_.6H_2_O, NaBH_4_, and NiCl_2_.6H_2_O, were supplied by Merck, Germany. The concentration of lead, divalent and total, was determined using an atomic absorption spectrometer (Varian SpectrAA 220, Germany).

### NZVI preparation

The iron nano particles were prepared on-site to prevent more oxidation of nZVI surface. To synthesize the nZVI particles, 0.15 M NaBH_4_ solution was added slowly with the rate of 1 to 2 mL/min into 0.1 M FeCl_3_.6H_2_O aqueous solution at ambient temperature and vigorously stirred at 400 rpm [12]. During this reaction, ferric ions were reduced into black particles by sodium borohydride as the reductant, according to the following reaction [13]:

1 $$ {{4\mathrm{F}\mathrm{e}}^{3+}}_{\left(\mathrm{a}\mathrm{q}\right)} + 3{\mathrm{BH}}_4 + 9{\mathrm{H}}_2\mathrm{O}\ \to\ {{4\mathrm{F}\mathrm{e}}^{\mathrm{o}}}_{\left(\mathrm{s}\right)}\downarrow + 3{\mathrm{H}}_2{\mathrm{BO}}_3 + {{12\mathrm{H}}^{+}}_{\left(\mathrm{a}\mathrm{q}\right)} + 6{\mathrm{H}}_{2\left(\mathrm{g}\right)}\uparrow $$

The black precipitates were filtered by vacuum filtration through 0.45 μm filter papers and then washed with DI water and ethanol three times.

### NZVI stabilization

Previous researches have indicated that nZVI particles aggregate quickly, after decreasing surface area for reaction and limiting mobility. To control nano particle agglomeration, various particle stabilizing strategies have been reported that surface modification with surfactant is one of the most important approaches [14]. Surfactants, such as starch, could be coated on existing nZVI particles in a post-synthesis process; or synthesizing nZVI in the presence of polymer in a pre-synthesis process. The post-synthesis stabilization approach has been shown to decrease reactivity whereas the pre-synthesis approach has improved reactivity and significantly increased surface area [15]. In the present study, nZVI was stabilized by starch in a pre-synthesis process, according to He and Zhao method [16], which is termed here as S-nZVI.

### Batch experiments

The batch experiments were conducted to find the finest condition for columns tests. In addition, useful information could be obtained about the effect of some key parameters. As listed in Table 1, the effects of several main variables were investigated in batch tests, one parameter changed while others were kept constant. Batch experiments were carried out in sealed flasks at 20°C. Predefined S-nZVI and 125 mL buffered Pb^2+^ solution with initial concentration of 200 mg/L and pH 4.0 were added to each flask, and the suspension was stirred. Aliquots of the samples were taken at certain time intervals and analyzed immediately following paper filtration. All experiments were conducted in duplicate and the results were averaged.

**Table 1:** Experimental design in present study* **all experiments were conducted in ambient temperature 15**–**20** **°C**

**Experimental set**			**Variable parameter**						**Controlled conditions**
Batch		1-1	Controlled pH	2.0	4.0	6.0	8.0		S-nZVI: 0.5 g/L, C_o_: 200 mg/L
		1-2	Initial Pb^2+^ (mg/L)	100	200	400	800		S-nZVI: 0.5 g/L, pH: 4.0
		1-3	S-nZVI con.(g/L)	0.1	0.2	0.5	1.0	2.0	C_o_: 200 mg/L, pH: 4.0
continuous experiments	Transparent column	2-1	Seepage velocity (m/d)	5	10	20	40		C_o_ = 200 mg/L, pH: 4, Seepage velocity: 10 m/d
		2-2	S-nZVI loading (g)	1	2	5	10		nZVI: 5 g, pH: 4, C_o_ = 200 mg/L
	Bench-scale	3-1	Seepage velocity (m/d)	10	20	40			nZVI: 15 g, pH: 4, C_o_ = 200 mg/L

### Continuous experiments

**Figure 1:**
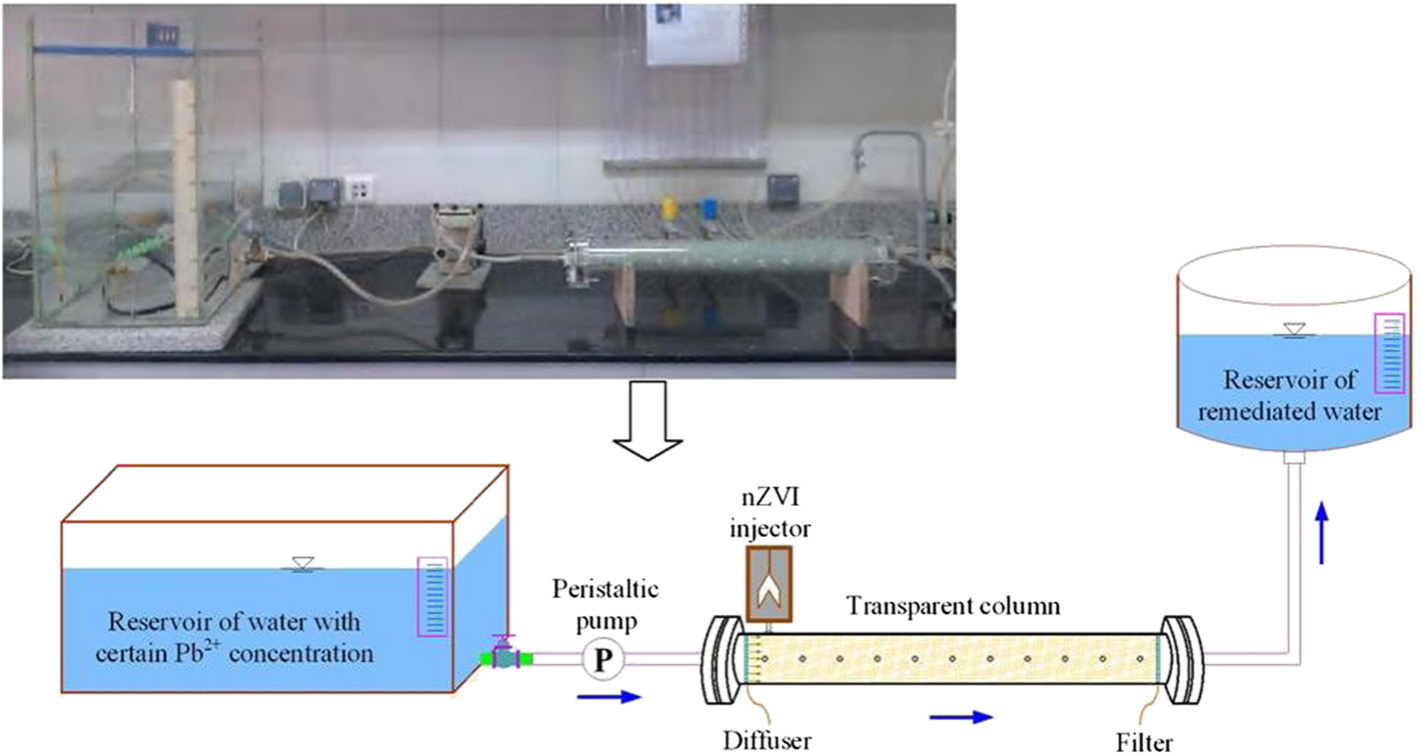
**Picture and schematic of transparent column model filled with glass beads.**
- 2 - bench-scale apparatus consisted of two PVC columns with 300 mm diameter and 1250 mm height filled with packed sand (Figure 2).

Continuous experiments were performed in two configurations:

- 1 - Transparent column with 55 mm diameter and 650 mm length filled with glass beads (Figure 1);

**Figure 2:**
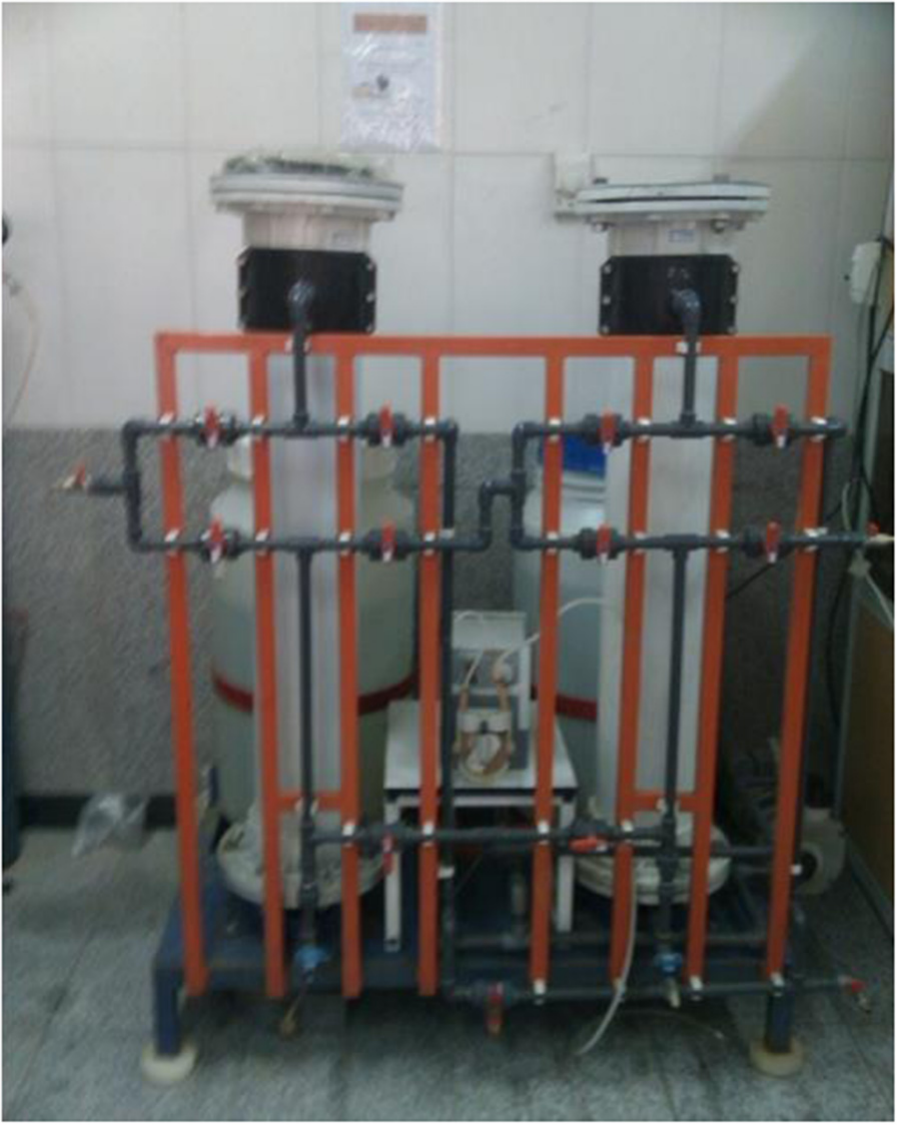
**Picture of bench**-**scale model.**

Prior to each run, in the first system, glass beads were soaked in hydrogen peroxide solution for 10 hr, washed with de-ionized water, and finally baked at 105°C for 24 hr [17]. For the continuous system, sands were prepared by baking at 500°C for 24 hr to eliminate adsorbed organic matter. In this set of experiments, the pH of the solution was adjusted to 4 ± 0.2 using 0.1 N HCl.

The effects of some flow parameters were investigated by transparent column model, including seepage velocity and S-nZVI loading. Seepage velocity tests, consisted of 5, 10, 20, and 40 m/d, were conducted by distilled water with initial Pb^2+^ concentrations of 200 mg/L, and 5 g S-nZVI injection. S-nZVI loading tests were experimented by initial Pb^2+^ concentrations of 200 mg/L, seepage velocity of 10 m/d, and frequent 5, 10, and 15 g S-nZVI injection.

The bench-scale experiments were planned based on the results of the batch and transparent column tests. The S-nZVI injected into the bottom center of the column forms a permeable reactive zone which reduced inlet pb^2+^. This configuration is applicable to study the effects of groundwater ionic strength, porous media type, seepage velocity, initial concentration of pb^2+^ and nano particle loading, at the same time. Three treatments, consisted of 5, 10, and 15 g initial S-nZVI loading, were carried out in the bench-scale model. Other conditions were kept as seepage velocity 10 m/d, pH 4, and were used wastewater with initial Pb^2+^ concentration of 200 mg/L.

## Results and discussion

### Characteristics of S-nZVI

**Figure 3:**
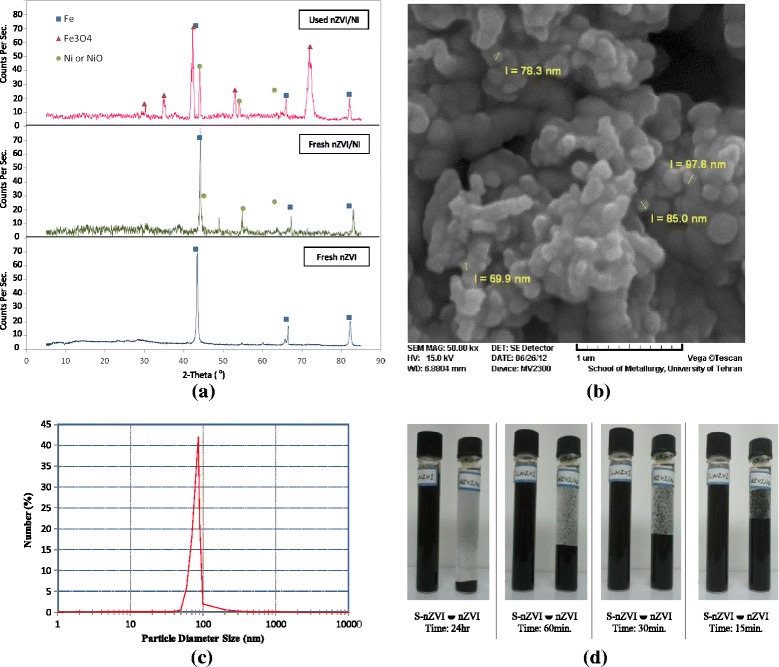
**Characteristic of synthesized nZVI, (a) XRD diagram, (b) SEM image, (c) DLS results, (d) stabilization status.**

To characterize the stabilized iron nano particles, X-ray powder diffraction (XRD), scanning electron microscopy (SEM), and dynamic light scattering (DLS) were recorded as the results shown in Figure 3. The starched nZVI particles displayed much less agglomeration than those prepared without a stabilizer while S-nZVI remained suspended in water for several hours, non-starched particles agglomerated and precipitated within minutes. XRD results, obtained by a D8 Advanced Bruker diffractometer, indicated the presence of Fe° (peaked at 2θ = 42, 67, 82), and Fe_2_O_3_ (2θ = 35, 53). SEM analyses, by a S4160 FE-SEM device, denoted that the S-nZVI were present as discrete particles as opposed to dendritic flocs for non-starched particles. DLS was performed using a S-red Badge model ZEN1600. The mean particle size was estimated to be 78 nm with a standard deviation of 14 nm, which translated to a surface area of at least 25 m^2^/g.

### Batch experiments

**a. Effect of solution pH**

Generally, pH of solution is an important factor for heavy metal ions removal by nZVI. It is widely accepted that low pH has a negative effect on metal adsorption by nZVI [18]. When pH is lower than zero point charge (pH_zpc_), the positive charge surface of nZVI will repulse metal cations, and results lower removal efficiency [19]. On the other hand, it is found out from equation 2 that H^+^ is strongly produced along the Pb^2+^ reduction reaction that point to an alkaline environment is preferred by the Pb^2+^ removal in aqueous solution [20].

2 $$ 2{\mathrm{Fe}}^{\mathrm{o}} + 3{\mathrm{Pb}}^{2+} + 4{\mathrm{H}}_2\mathrm{O}\to 2\mathrm{FeOH} + 3{\mathrm{Pb}}^{\mathrm{o}} + 2{\mathrm{H}}^{+} $$

**Figure 4:**
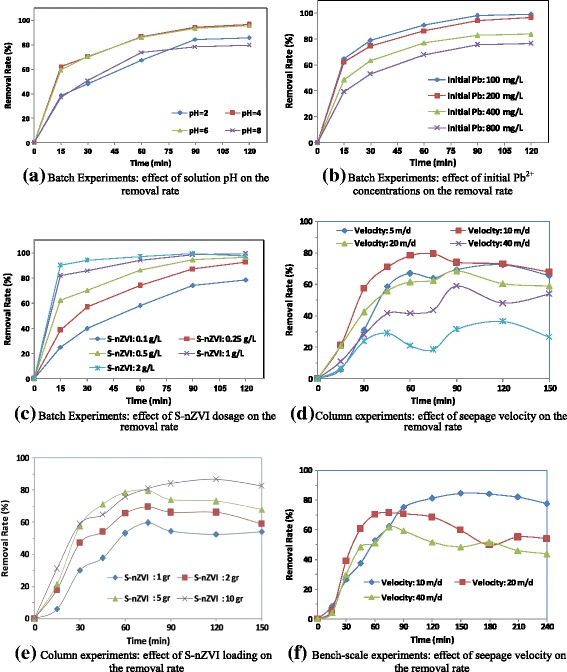
**Results of Pb** ^**2+**^ **removal by experiments in batch, column and bench-** **scale models.**

Figure 4a presents the results of batch experiments in which S-nZVI are exposed to Pb^2+^ buffered solution with different pH values. At pH <4.0 or >6.0 after 60 min, less than 60% of Pb^2+^ was degraded, while more than 80% removal efficiency was obtained after 60 min at pH 5 ± 1. The results suggest that rapid reduction of Pb^2+^ into Pb^o^ occurs while pH < 6, and adsorption was optimized by adjusting the pH > 4. Thus, it can be concluded that pH around 5 favors for Pb^2+^ removal by nZVI.

**b. Effect of initial Pb**^**2+**^**concentration**

**Figure 5:**
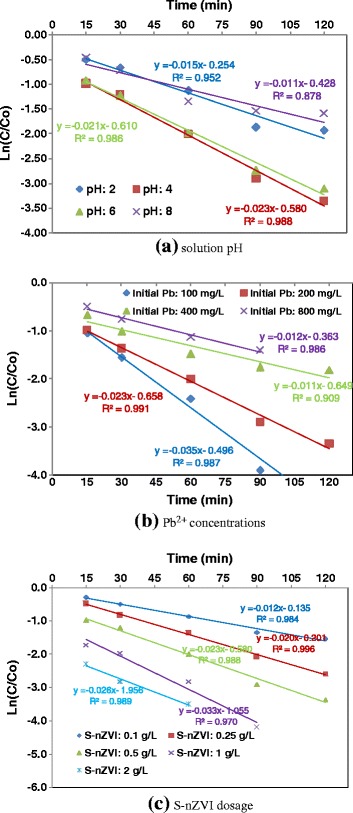
**Pseudo first-order adsorption kinetic plots (corresponding to Figure** **4** **a, b and c, respectively).**

As shown in Figure 4b, the effect of initial concentration of Pb^2+^ on removal efficiency was investigated in the range of 100 to 800 mg/L. The equilibrium time became longer and the final removal efficiency of Pb^2+^ decreased as the initial Pb^2+^ concentration increased; So, the percentage of Pb^2+^ removed within 60 min at an initial concentration of 100 mg/L was nearly 85%, and it was only 58% at an initial concentration of 800 mg/L. Furthermore, as revealed in Figure 5b, the plot well fitted to the pseudo first order adsorption model, where the observed rate constant decreased significantly as the initial Pb^2+^ concentration increased. Generally, when nZVI concentration was constant, the lower efficiency and slower rate of Pb^2+^ removal were found at higher initial concentrations of Pb^2+^.These results suggest that the capacity of the S–nZVI for lead removal is about 430 mg Pb/g S-nZVI.

**c. Effect of S-nZVI concentration**

As shown in Figure 4c, the Pb^2+^ removal efficiency improved as the S-nZVI concentration increased. The removal efficiency of Pb^2+^ was about 50% using S-nZVI at 0.1 g/L for 60 min, but was nearly 95% when the S-nZVI concentration was higher than 1 g/L. In the same conditions, K_obs_ raised as the S-nZVI concentration increased. These phenomena can be attributed to the increase in available active sites resulting from the increase in S-nZVI concentration, where the lead reduction occurred. Additionally, lead ions removal sharply enhanced by increasing contact time for the first 60 min, and then gradually approached equilibrium after approximately 120 min.

**d. Kinetics of the Pb**^**2+**^**reduction**

The adsorption kinetics of Pb^2+^ ions was studied to determine the required time to achieve equilibrium adsorption of lead ions on the adsorbents. It was reported that nZVI can remove metal ions from aqueous solutions by various mechanisms, including electrostatic adsorption, complex formation, reduction, and precipitation [21]. It seems that when nZVI was used, the nano particles captured aqueous lead ions easily and rapidly because of their large surface areas and high reactivity.

In this research, the kinetics was investigated experimentally under different values of initial Pb^2+^ concentration, solution pH, and S-nZVI dosage by using pseudo first-order reaction model that can be represented as following:

3 $$ \ln \left(C/{C}_o\right)=-{K}_{obs}t $$

where *C*_*o*_ and *C* are the concentration of Pb^2+^ (mg/g) at initial and time *t* (min), respectively. *K*_*obs*_ (min^−1^) is the equilibrium rate constant for first order adsorption. Therefore, by plotting *ln*(*C*/*C*_*o*_) against *t*, the values of *K*_*obs*_ can be found from the slope of the revealed plots.

Several studies have investigated the kinetic of Pb^2+^ removal by nZVI. Among a number of kinetic models, such as pseudo zero-order, pseudo first-order and pseudo second-order kinetic models, the pseudo first-order kinetic model was the most suitable which approves the best fit with the experimental data of Pb^2+^ removal compared to the rest of models [2]. As seen in Figure 5, the experimental data were well fitted to the pseudo first-order adsorption model with the high correlation coefficients. The parameters obtained by linear regression analysis were offered in Table 2. The results indicated a decrease in *K*_*obs*_ values from 0.04 to 0.01 g/mg/min, when initial Pb^2+^ concentration increased from 100 to 800 mg/L. The rate constant *K*_*obs*_ also increased by increasing S-nZVI dosage, and by approaching to a solution pH of 5.

**Table 2:** **Pseudo first**-**order adsorption kinetics constants for Pb** ^**2**+^ **removal by S**-**nZVI**

**Parameters**	**pH:** **2.0**	**4.0**	**6.0**	**8.0**	
K_obs_ (1/min)	−0.015	−0.024	−0.022	−0.011	
R^2^	1.0	0.999	0.998	0.991	
Fixed conditions: Initial Pb^2+^ 200 mg/L, S-nZVI 0.5 g/L					
**Parameter**	**Initial Pb** ^**2+**^ **:** **100 mg/** **L**	**200**	**400**	**800**	
K_obs_ (1/min)	−0.035	−0.023	−0.011	−0.012	
R^2^	0.999	0.999	0.999	0.997	
Fixed conditions: S-nZVI 0.5 g/L, pH 4.0,					
**Parameter**	**S**-**nZVI**:**0.1 g/** **L**	**0.25**	**0.5**	**1.0**	**2.0**
K_obs_ (1/min)	−0.012	−0.020	−0.024	−0.033	−0.026
R^2^	0.999	0.981	0.999	1.0	1.0
Fixed conditions: Initial Pb^2+^ 200 mg/L, pH 4.0,					

**e. Adsorption isotherms**

Experimental data were modeled using the well known adsorption models described by the Freundlich and Langmuir equations to study the ability of Pb^2+^ ions to adsorb on S-nZVI [22].

Based on the results of our experimental data fitting on these isotherms, in Table 3, nZVI has the good ability to reduce Pb^2+^ to Pb^o^ which can then be absorbed by the nZVI easily. Furthermore, data fitting by using the Langmuir model give a better fit than by using the Freundlich model. This can be seen from the fitting data obtained that the correlation coefficient is higher for the Langmuir adsorption isotherm (~0.92) compared to that of the Freundlich model (~0.84).

**Table 3:** **Freundlich and Langmuir adsorption isotherms constants for Pb** ^**2**+^ **removal by S**-**nZVI**

**Parameters**	**pH**:**2.0**		**4.0**	**6.0**	**8.0**	
Freundlich	1/n	−0.51	−0.18	−0.21	−0.65	
	K_n_	1996	571	629	3662	
Langmuir	q_max_	0.14	0.24	0.23	0.12	
	K_l_	0.05	0.21	0.16	0.04	
Fixed conditions: Initial Pb^2+^ 200 mg/L, S-nZVI 0.2 g/L						
**Parameter**		**Initial Pb** ^**2**+^: **100 mg**/**L**	**200**	**400**	**800**	
Freundlich	1/n	−0.11	−0.17	−0.45	−0.67	
	K_n_	208	564	4615	43143	
Langmuir	q_max_	0.13	0.24	0.33	0.50	
	K_l_	0.71	0.20	0.03	0.01	
Fixed conditions: S-nZVI 0.2 g/L, pH 4.0,						
**Parameter**		**S**-**nZVI**:**0.1 g**/**L**	**0.25**	**0.5**	**1.0**	**2.0**
Freundlich	1/n	−0.85	−0.39	−0.18	−0.05	−0.03
	K_n_	43228	2335	571	204	38
Langmuir	q_max_	0.42	0.29	0.24	0.16	0.09
	K_l_	0.02	0.06	0.21	1.43	2.83
Fixed conditions: Initial Pb^2+^ 200 mg/L, pH 4.0,						

### Continuous experiments

#### Transparent column

**a. Effect of seepage velocity**

The results of seepage velocity tests are shown in Figure 4c. It was observed that a seepage velocity of 10 m/d yielded the maximum removal rate. Furthermore, any variation, increasing or decreasing of this velocity, had a negative effect on the Pb^2+^ removal. Higher seepage velocities enhanced the mobility of nano particles through porous media and reduced the contact time and as a result, reduced the remediation efficiency.

**b. Effect of S-nZVI loading**

As shown in Figure 4e, when S-nZVI loading rose from 1 to 10 g, the Pb^2+^ removal efficiency within 60 min increased from about 50% to 80%. It is found out that when further amount of S-nZVI was injected, the Pb^2+^ removal has been improved.

### Bench-scale model

The results of bench-scale experiments, illustrated in Figure 4f, indicated that the removal efficiency through sand materials were higher than glass beads. In addition, increasing seepage velocity had a decreasing effect on the Pb^2+^ remediation. Through bench-scale experiments, the best Pb^2+^ remediation efficiency was achieved, i.e. about 81%. In pH of 4, initial concentration of 200 mg/L, and 15 g S-nZVI injection, finally 31 L of treated water with Pb^2+^ concentration less than 20 mg/L was acquired. Therefore, it could be proposed that the capacity of S-nZVI for *in*-*situ* lead removal is about 300 mg Pb^2+^/g S-nZVI.

## Conclusions

Findings of this study indicated that starched nZVI has a good feasibility for *in*-*situ* lead remediation in contaminated water. Batch experiments proved that pH of solution was an important parameter while kinetics coefficients were directly related to pH with correlation coefficients R^2^ > 0.90. In addition, increasing of S-nZVI dosage or decreasing Pb^2+^ initial concentration, both lead to enhancement in removal efficiency. It means that if mass-ratio between nZVI and Pb is kept constant, i.e. about 2.5, the removal rate would be invariable.

Transparent column experiments revealed that Pb^2+^ remediation also was mostly influenced by seepage velocity, grain size, and type of porous media. Bench-scale results confirmed the batch and transparent column outcomes. As a final point, because of the fast reaction kinetics and high Pb^2+^ removal capacity, S-nZVI has the fine potential to become an effective remedial agent in PRB for *in*-*situ* immobilization of lead in polluted groundwater resources.

## Abbreviations

nZVI: nano Zero Valent Iron particles
S-nZVI: Stabilized nano Zero Valent Iron particles
U.S.EPA: U.S. Environmental Protection Agency
PRB: Permeable Reactive Barriers
XRD: X-Ray powder Diffraction
SEM: Scanning Electron Microscopy
DLS: Dynamic light scattering
